# Numerical validation data of tensile stress zones and crack zones in bamboo reinforced concrete beams using the Fortran PowerStation 4.0 program

**DOI:** 10.1016/j.dib.2020.105332

**Published:** 2020-02-26

**Authors:** Muhtar

**Affiliations:** Department of Civil Engineering, Faculty of Engineering, University of Muhammadiyah Jember, Jember, 68121, Indonesia

**Keywords:** Numerical validation, Finite element method, Tensile stress zone, Crack zone, Bamboo reinforced concrete

## Abstract

Numerical verification is carried out in order to control the compatibility of the BRC beam crack pattern with the stress contour at the ultimate load. The numerical method used is the finite element method (FEM) using the Fortran PowerStation 4.0 program. Material data entered is the elasticity modulus (*E*) and Poisson's ratio (*ν*). Ultimate load input data is taken from BRC beam testing in the laboratory. Bamboo reinforcement and concrete are considered to have the same displacement with a different elasticity modulus (*E*), so they experience different stresses. The triangle element is employed to model the plane-stress with two directions of displacement at each nodal point, so that each element has six degrees of freedom. The BRC beam tensile stress data from the Fortran PowerStation 4.0 program is processed into a tensile stress data table and becomes the Surfer program input data for mapping tensile stress zone images. Crack pattern data from laboratory beam testing is processed into crack zone pattern photo data and then compared to the tensile stress zone images. From the image data of the tensile stress zones and the crack zones of the BRC beam have compatibility. The Fortran PowerStation 4.0 programming language data in this article can be used for further research with the discretization of triangular elements in other cases. This article consists of a data table, a picture of a crack pattern zone, a drawing of tensile stress zones, and photo documentation. The data is related to “Enhancing bamboo reinforcement using a hose-clamp to increase bond-stress and slip resistance” [1].

**Table:** Specifications Table

Subject	Engineering.
Specific subject area	Civil and structural engineering.
Type of data	Table, image, program.
How data were acquired	The crack pattern data was obtained from the beam flexural test (Fig. 15). The stress contour data was obtained from FEM analysis, using the Fortran PowerStation 4.0 and Surfer programs. Crack pattern data from the beam flexural test is processed and analyzed into crack zone image data. Stress data from FEM analysis is processed into stress table data and becomes the input data for the Surfer program. Data from the Surfer program is processed into stress zone image data. Then, all data is processed, compared, and analyzed into table data, cracks pattern zone image data, tensile stress zone image data, and photo data.
Data format	Raw and analyzed.
Parameters for data collection	Crack pattern data and maximum tensile stress data are two highly related data, in which cracks will occur at the maximum tensile stress position. The initial cracks until the collapsed beams are obtained through observation with a crack detector under a gradually increasing load. Crack pattern zone data from the laboratory beam test needs to be validated by other methods to determine compatibility with stresses that occur. Stress data and stress zone images data are obtained through FEM analysis using the Fortran PowerStation 4.0 and the Surfer programs. The Fortran PowerStation 4.0 programming language can be used for further research.
Description of data collection	The crack pattern data was collected through beam testing in the laboratory. Initial crack and subsequent crack data up to the beam collapsing are obtained through observation in stages, according to the beam loading stage. The crack detector is used to observe cracks. Each crack is numbered and drawn as the crack line. Then the crack data is processed and documentation taken, with results termed the crack zone image data. Tensile stress zone data was obtained from two sources, FEM analysis using the Fortran PowerStation 4.0 and the Surfer programs. FEM analysis with the Fortran PowerStation 4.0 program was obtained for direction stresses of X, Y, and Z. The X directional stress is tensile stress that causes cracks. The X direction stress data is then transferred to the Surfer program to generate the stress zone contour image data. The crack pattern data and the stress zone contour image data are compared and analyzed into table data, image data, program data, and photo data, all of which are termed intact data. This intact data was obtained from two specimens, namely a BRC beam and an SRC beam, to obtain crack patterns and tensile stresses with different reinforcement materials. The behaviors of the crack pattern and the stress zone from the two beams can be used as basis for further research.
Data source location	University of Muhammadiyah Jember, Jember, 68,121, Indonesia, and University of Brawijaya, Malang 65,145, Indonesia
Data accessibility	Data with the article, raw data can be found in Table 1, Table 2, Table 3, http://bit.ly/351FPqU, http://bit.ly/2MBqas9, http://bit.ly/2F17w8F, http://bit.ly/2rDPeaI, http://bit.ly/2Q4Ihc1, http://bit.ly/2MTh22j, http://bit.ly/2ZvZWMU, http://bit.ly/2u2K2xR, http://bit.ly/2ZybLCd, and http://bit.ly/2Q7j2Wp
Related research article	Enhancing bamboo reinforcement using a hose-clamp to increase bond-stress and slip resistance. https://doi.org/10.1016/j.jobe.2019.100896 [1]

**Table:** 

**Value of the Data** • This data is useful for researchers in developing bamboo reinforced concrete structures, especially for simple construction in areas of abundant bamboo. • Data can be used for further insight and development, especially stress analysis, capacity, and behavior of bamboo reinforced concrete beams with strengthening reinforcement. • This data contains a program that can be used as a reference in analyzing and calculating stresses of the BRC beam and SRC beam by triangular element discretizing. • The added value of this data is in the programming language; Fortran PowerStation 4.0 can now be used generally in further research to analyze the displacement and stress of two-dimensional plane-stress elements.

## 1 Data

**Fig. 1:**
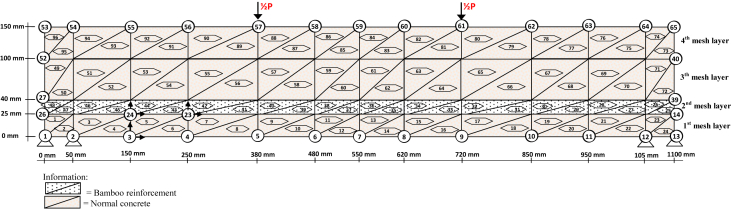
The discretization of the BRC beam using the triangle element [1].

**Fig. 2:**
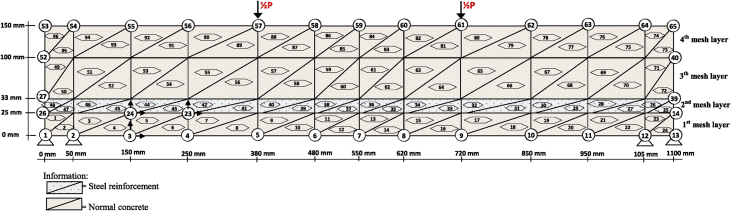
The discretization of the SRC beam using the triangle element.

The discretization image data of the BRC beam and SRC beam with triangular elements is shown in Fig. 1 and Fig. 2. The reduction data of the stiffness of the BRC beam and the SRC beam after the initial crack occurs up until the beam collapses is shown in Table 1 and Table 2. The input data for the Fortran PowerStation 4.0 program for the BRC beam is shown in the following link: http://bit.ly/351FPqU, and the input data for the SRC beam is shown in the link: http://bit.ly/2MBqas9. The programming language data for the Fortran PowerStation 4.0 program with the discretization of triangular elements is shown in the link: http://bit.ly/2F17w8F.

**Table 1:** The reduction data of the stiffness of the BRC beam after initial cracking occurs up until the ultimate load [2].

Layer number	Modulus of elasticity (*E*) of the BRC beam										
	Elastic condition	Plastic conditions with gradual loads									
	0–8.5 kN	9 kN	11 kN	15 kN	17 kN	21 kN	23 kN	25 kN	27 kN	29 kN	33 kN
4th mesh layer	268,512,89	161,107,73	161,107,73	161,107,73	161,107,73	161,107,73	161,107,73	161,107,73	120,830,80	112,775,41	85,924,12
3rd mesh layer	268,512,89	161,107,73	161,107,73	161,107,73	161,107,73	161,107,73	161,107,73	120,830,80	107,405,16	93,979,51	75,183,61
2nd mesh layer	247,451,73	138,845,32	115,704,43	115,704,43	115,704,43	104,133,99	104,133,99	104,133,99	69,422,66	69,422,66	55,538,13
1st mesh layer	268,512,89	134,256,45	118,145,67	83,239,00	67,128,22	51,017,45	51,017,45	37,591,80	32,221,55	26,851,29	13,291,39

**Table 2:** The reduction data of the stiffness of the SRC beam after initial cracking occurs up until the ultimate load [2].

Layer number	Modulus of elasticity (*E*) of the SRC beam										
	Elastic condition	Plastic conditions with gradual loads									
	0–9 kN	10 kN	11 kN	12 kN	13 kN	15 kN	17 kN	19 kN	21 kN	23 kN	24 kN
4th mesh layer	268,512,89	268,512,89	201,384,67	201,384,67	201,384,67	201,384,67	201,384,67	187,959,02	187,959,02	134,256,45	114,117,98
3rd mesh layer	268,512,89	268,512,89	201,384,67	201,384,67	187,959,02	187,959,02	187,959,02	174,533,38	174,533,38	134,256,45	114,117,98
2nd mesh layer	407,825,73	432,093,18	324,069,88	324,069,88	302,465,22	302,465,22	280,860,56	280,860,56	259,255,91	216,046,59	183,639,60
1st mesh layer	268,512,89	268,512,89	201,384,67	201,384,67	187,959,02	187,959,02	174,533,38	161,107,73	147,682,09	134,256,45	120,830,80

**Fig. 3:**
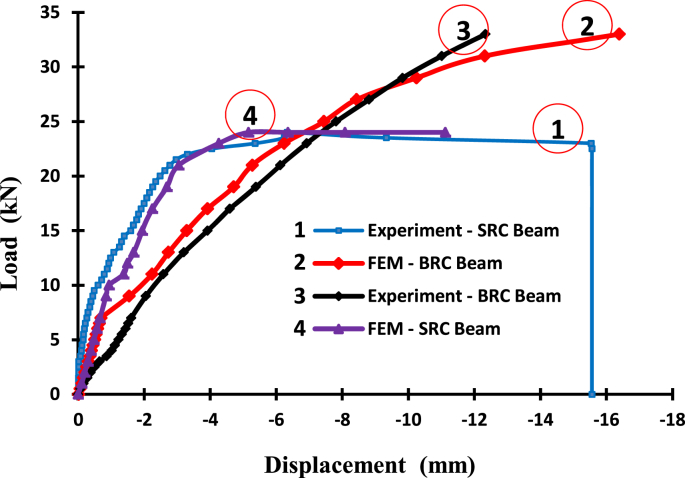
The load-displacement relationship of the BRC and SRC beams with experiment and FEM.

The data of the load-displacement relationship of the BRC beam and SRC beam from experiments and FEM analyses is shown in Table 3, while the image data of the load-displacement relationship diagrams from experiments and FEM analyses is shown in Fig. 3. The displacement contours data in the X-direction and Y-direction of the Surfer program for the BRC beam is shown in Fig. 8 and Fig. 9, and the displacement contour data in the X-direction and Y-direction from the Surfer program for the SRC beam is shown in Fig. 12 and Fig. 13.

**Table 3:** The data of load and displacement of BRC beams and SRC beams.

The data of load and displacement of BRC beams and SRC beams							
Experiment–BRC Beam		Experiment–SRC Beam		FEM–BRC Beam		FEM–SRC Beam	
Load (kN)	Displacement (mm)	Load (kN)	Displacement (mm)	Load (kN)	Displacement (mm)	Load (kN)	Displacement (mm)
0,00	0,00	0,00	0,00	0,00	−0,00	0,00	0,00
0,50	−0,07	1,00	−0,01	0,50	−0,07	1,00	−0,11
1,00	−0,17	2,00	−0,02	1,00	−0,12	2,00	−0,20
1,50	−0,28	4,00	−0,10	1,50	−0,16	3,00	−0,29
2,00	−0,38	5,00	−0,15	2,00	−0,21	4,00	−0,39
2,50	−0,51	6,00	−0,19	2,50	−0,26	5,00	−0,48
3,00	−0,65	7,00	−0,26	3,00	−0,31	6,00	−0,57
3,50	−0,86	8,00	−0,35	3,50	−0,36	7,00	−0,66
4,00	−1,02	9,00	−0,44	4,00	−0,41	9,00	−0,85
4,50	−1,12	10,00	−0,60	4,50	−0,45	10,00	−0,94
5,00	−1,22	11,00	−0,79	5,00	−0,50	11,00	−1,37
5,50	−1,33	12,00	−0,93	5,50	−0,55	12,00	−1,49
6,00	−1,44	13,00	−1,08	6,00	−0,60	13,00	−1,69
6,50	−1,52	14,00	−1,31	6,50	−0,65	15,00	−1,94
7,00	−1,61	15,00	−1,59	7,00	−0,70	17,00	−2,25
9,00	−2,05	16,00	−1,77	9,00	−1,55	19,00	−2,69
11,00	−2,59	17,00	−1,91	11,00	−2,24	21,00	−3,05
13,00	−3,20	18,00	−2,08	13,00	−2,74	23,00	−4,26
15,00	−3,93	19,00	−2,26	15,00	−3,29	24,00	−5,16
17,00	−4,59	20,00	−2,48	17,00	−3,92	24,00	−6,35
19,00	−5,39	21,00	−2,78	19,00	−4,71	24,00	−8,09
21,00	−6,13	22,00	−3,31	21,00	−5,28	24,00	−11,12
23,00	−6,93	23,00	−5,36	23,00	−6,24		
25,00	−7,81	24,00	−6,33	25,00	−7,45		
27,00	−8,81	23,50	−9,33	27,00	−8,43		
29,00	−9,83	23,00	−15,54	29,00	−10,25		
31,00	−11,01	22,50	−15,56	31,00	−12,32		
33,00	−12,34	0,00	−15,56	33,00	−16,39

**Fig. 4:**
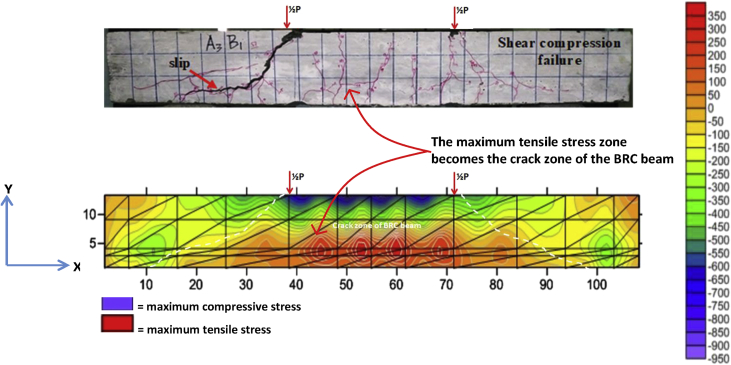
The data validation of crack pattern with tensile stress contours of the BRC beam.

**Fig. 5:**
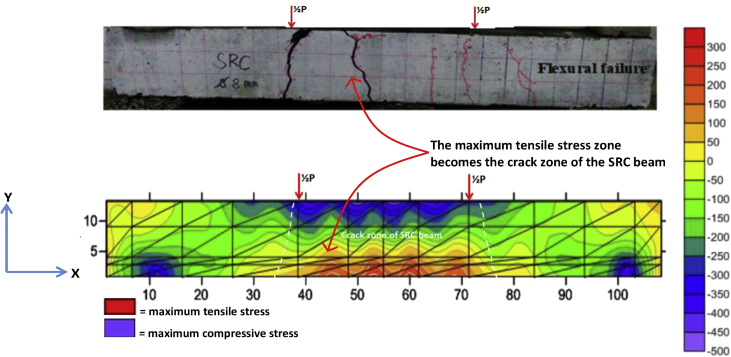
The data validation of crack pattern with tensile stress contours of the SRC beam.

**Fig. 6:**
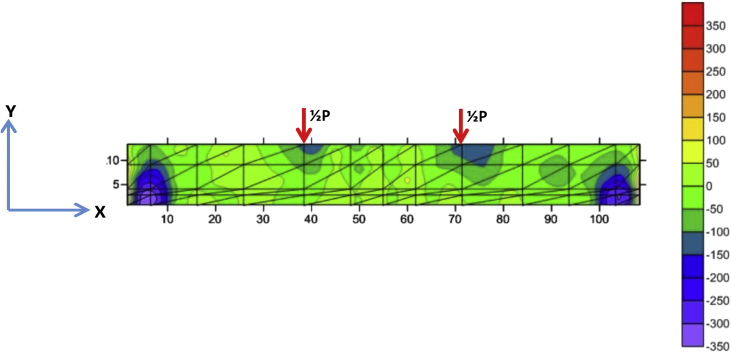
The stress of Y-direction of BRC beam.

**Fig. 7:**
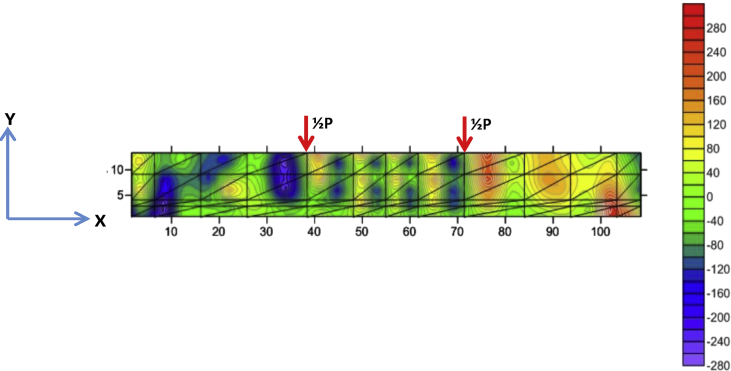
The stress of XY-direction of BRC beam.

**Fig. 8:**
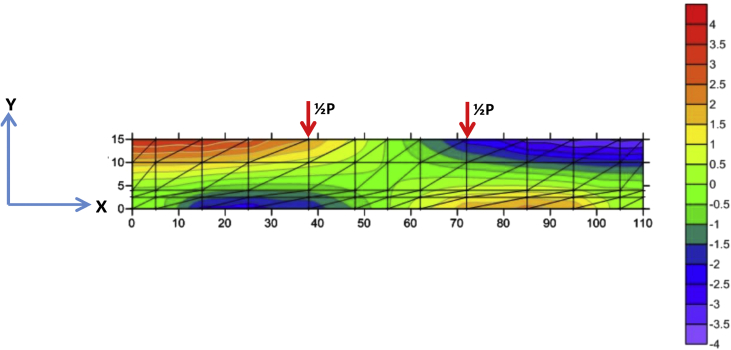
The displacement of X-direction of BRC beam.

**Fig. 9:**
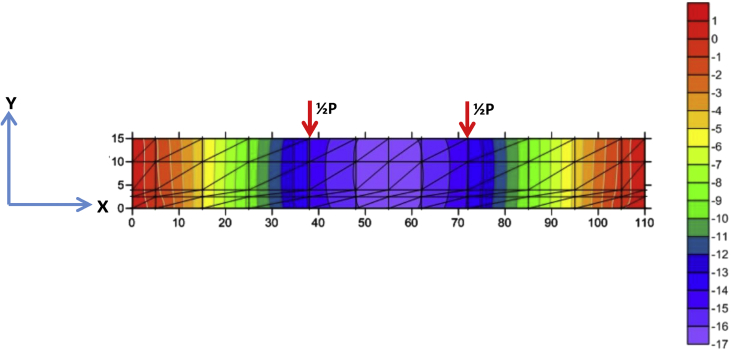
The displacement of Y-direction of BRC beam.

**Fig. 10:**
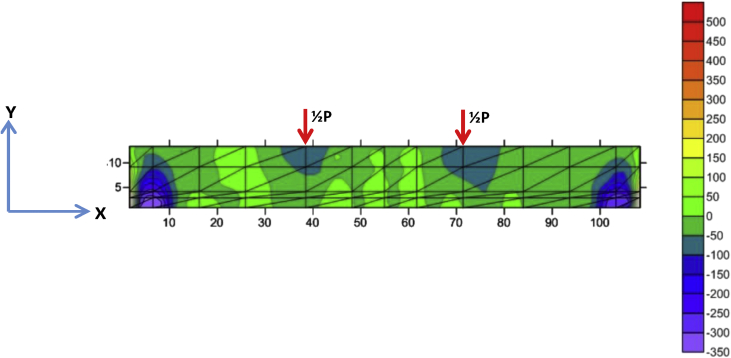
The stress of Y-direction of SRC beam.

**Fig. 11:**
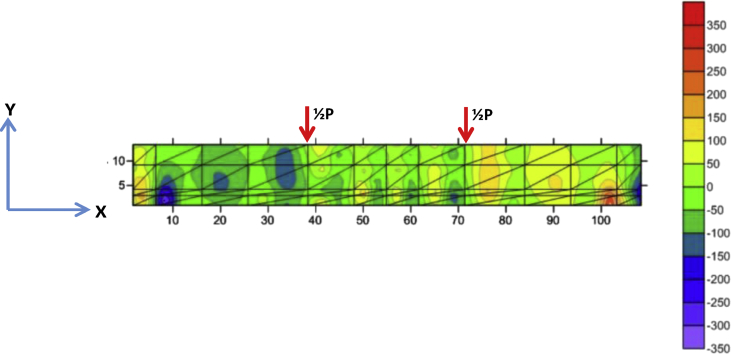
The stress of XY-direction of SRC beam.

**Fig. 12:**
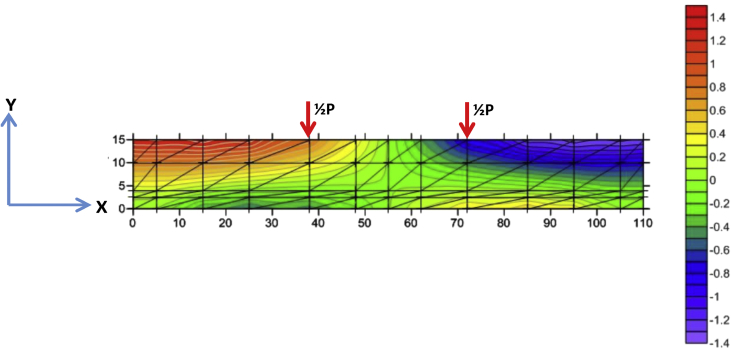
The displacement of X-direction of SRC beam.

**Fig. 13:**
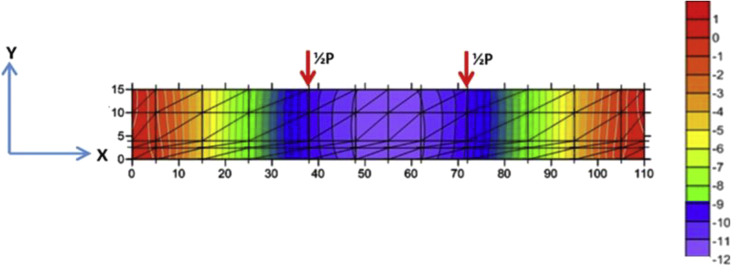
The displacement of Y-direction of SRC beam.

The table data of stress on the X-direction, Y-direction, and XY-direction from FEM analysis for the BRC beam is shown in the following link: http://bit.ly/2rDPeaI and the stress contour image data from the Surfer program is shown in Figs. 4, Fig. 6, and Fig. 7. The table data of stress on the X-direction, Y-direction, and XY-direction from FEM analysis for the SRC beam is shown in the following link: http://bit.ly/2Q4Ihc1, while the stress contour image data from the Surfer program is shown in Figs. 5, Fig. 10, and Fig. 11. Photographs of crack pattern data and tensile stress contour data for the analysis of the compatibility of the zone are shown in Figs. 4 and 5.

## 2 Experimental design, materials, and methods

Numerical analysis was carried out with 2D and the experiments with 3D as shown in Fig. 15. Data from 2D numerical analysis obtained data of X-direction stress, Y-direction stress, XY-direction stress, X-direction deflection, and Y-direction deflection. While the data from the experiments only obtained data on crack patterns, loads, strain, and deflection. So that the validation of both focuses on X-direction stress or tensile stress that causes cracking and Y-direction deflection. The validation of the tensile stress zone and crack patterns zone is shown in Figs. 4 and 5 and the validation of deflection are shown in Table 3, Figs. 9, and Fig. 13. The validation of Y-direction stress, XY-direction stress, and X-direction deflection are not done because experimental data are not obtained.

**Fig. 14:**
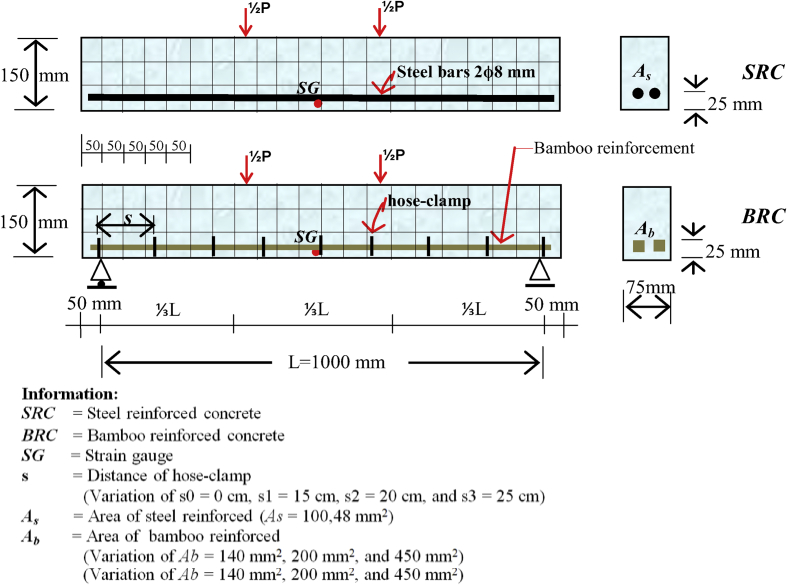
Detail and geometry of the bamboo reinforced concrete beam [1,4].

**Fig. 15:**
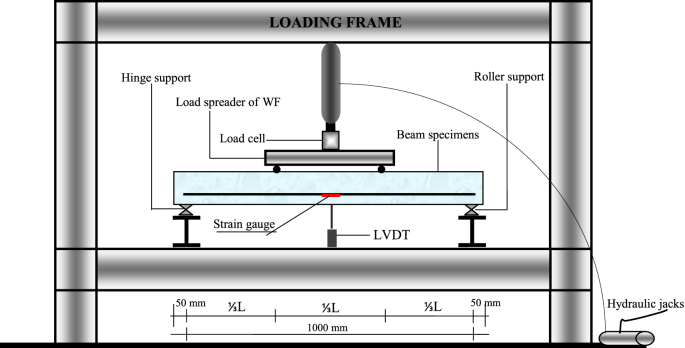
Flexural test settings for the four-point flexural test method [1,[4], [5], [6]].

Data validation between laboratory data and numerical analysis is carried out through a series of activities, namely beam flexural testing in the laboratory, numerical analysis with finite element method (FEM), program simulation with Fortran PowerStation 4.0, and simulation with the Surfer program. Activities in the laboratory are flexural tests of the BRC beam and the SRC beam to obtain data on crack patterns, collapse patterns, and ultimate loads. The test settings for the BRC beam and the SRC beam are shown in Fig. 15. The geometry and details of the reinforcement of BRC beams and SRC beams are shown in Fig. 14.

The material data of bamboo, steel, and concrete consists of Modulus of elasticity (*E*) and Poisson's ratio (*ν*). The modulus of elasticity of bamboo (*E*_*b*_) is 17,235.74 MPa with Poisson's ratio (*ν*_*b*_) of 0.25. The modulus of elasticity of concrete (*E*_*c*_) is 26,299.01 MPa with Poisson's ratio (*ν*_*c*_) of 0.20. The modulus of elasticity of steel (*E*_*s*_) is 207,735.92 MPa with Poisson's ratio (*ν*_*s*_) of 0.3.

The constitutive relationship analysis of the finite element method employs plane-stress theory. The triangle element is used to model the plane-stress element with two main displacement directions at each nodal point, so that the element has six degrees of freedom. The discretization of the beam plane using the triangular element is shown in Figs. 1 and 2. Modulus of elasticity (*E*) for each layer is calculated according to material conditions. Layers consisting of concrete and bamboo reinforcement are calculated using Eq. (1) [1], and for layers consisting of concrete and steel using Eq. (2) [3]. The solution to the plane-stress problem in the BRC beam and SRC beam is based on the stress-strain relationship as shown in Eq. (3) [1]. The main stresses on the BRC beam and SRC beam are calculated using Eq. (4) [1].

(1) $$E_{e}=E_{b}.V_{b}+E_{c}.V_{c}$$

(2) $$E_{e}=E_{s}.V_{s}+E_{c}.V_{c}$$

(3) $$\left\{\begin{array}{c} \sigma_{x}\\ \sigma_{y}\\ \tau_{xy} \end{array}\right\}=\frac{E}{\left(1+\nu^{2}\right)}\left[\begin{array}{ccc} 1 & \nu & 0\\ \nu & 1 & 0\\ 0 & 0 & \frac{1-\nu }{2} \end{array}\right]\left\{\begin{array}{c} \varepsilon_{x}\\ \varepsilon_{y}\\ \gamma_{xy} \end{array}\right\}$$

(4) $$\sigma_{1,2}=\frac{\sigma_{x}+\sigma_{y}}{2}\pm \sqrt{{\left(\frac{\sigma_{x}-\sigma_{y}}{2}\right)}^{2}+{\tau_{xy}}^{2}}=\sigma_{\max}$$

The steps for compiling the Fortran PowerStation 4.0 program data to get the beam tensile stress contour data are summarized as follows:

Step 1 Discretization of the plane of the BRC beam and the SRC beam with the discretization of the triangular element, as shown in Fig. 1, Fig. 2.

Step 2 Numbering of the triangular elements and the nodal points, as shown in Fig. 1, Fig. 2.

Step 3 Collection and calculation of the geometry data and the beam material data, such as modulus of elasticity of materials (*E*), Poisson ratio (*ν*), etc.

Step 4 Writing the programming language for the Fortran PowerStation 4.0 program for the triangular element, as shown in the following link: http://bit.ly/2F17w8F.

Step 5 Opening the Fortran PowerStation 4.0 program. As an example, the front view in the Fortran PowerStation 4.0 program is shown in the following link: http://bit.ly/2MTh22j.

Step 6 Writing programming language data (Step 4) in the Fortran PowerStation 4.0 program. As an example, a display of programming language is shown in the following link: http://bit.ly/2ZvZWMU.

Step 7 The Input DATA.DAT of the BRC beam and SRC beam in the Fortran PowerStation 4.0 program. The input data is shown in the following links: http://bit.ly/351FPqU and http://bit.ly/2MBqas9. An example of the input data display is shown in the following link: http://bit.ly/2u2K2xR.

Step 8 Running and processing the program analysis until there are no warnings and errors. If there are warnings and errors, check and correct the program data and input data.

Step 9 Downloading stress data X-direction, Y-direction, and XY-direction. Stress data is shown in the following link: http://bit.ly/2rDPeaI for the BRC beam stress, and http://bit.ly/2Q4Ihc1 for the SRC beam stress. For example, the display of stress data from the Fortran PowerStation 4.0 program is shown in the following link: http://bit.ly/2ZybLCd.

Step 10 Downloading displacement data X-direction and Y-direction. The displacement data for the BRC beam and SRC beam is shown in Table 3. An example of the displacement data display from the Fortran PowerStation 4.0 program is shown in the following link: http://bit.ly/2Q7j2Wp.

Step 11 Inputting stress data and displacement data on the Surfer program, running the program, and obtaining stress contour images data and displacement. Image data of stress contours and displacement are shown in Fig. 4, Fig. 5, Fig. 6, Fig. 7, Fig. 8, Fig. 9, Fig. 10, Fig. 11, Fig. 12, Fig. 13.

Step 12 Validation of drawing data for tensile stress contours (X-direction stress) with beam crack patterns from laboratory tests.

Step 13 Obtaining crack zone image data and tensile stress zone contour data. Image data of crack zones and contour zones of tensile stress are shown in Fig. 4, Fig. 5.

## 3 List of symbols

***E***: Modulus of elasticity
***E***_***e***_: The equivalent elasticity modulus of BRC beam or SRC beam
***E***_***b***_: Modulus of elasticity of bamboo reinforcement
***E***_***c***_: Modulus of elasticity of concrete
***E***_***s***_: Modulus of elasticity of steel reinforcement
***V***_***b***_: The relative volume of bamboo reinforcement in the calculated layer
***V***_***c***_: The relative volume of concrete in the calculated layer
***V***_***s***_: The relative volume of steel reinforcement in the calculated layer
***σ***_***x***_: Stress of X-direction
***σ***_***y***_: Stress of Y-direction
***σ***_***1,2***_: Main stress
***τ***_***xy***_: Shear stress of XY-direction
***ν***: Poisson's ratio
***ε***_***x***_: Strain of X-direction
***ε***_***y***_: Strain of Y-direction
***ϒ***_***xy***_: Shear strain of XY-direction

## References

[1] Muhtar, Dewi S.M., Wisnumurti, Munawir A. (2019). Enhancing bamboo reinforcement using a hose-clamp to increase bond- stress and slip resistance. Journal of Building Engineering.

[2] ACI Committee 318 (2014). Building Code Requirements for Structural Concrete (ACI 318M-14).

[3] Avram C., Facaoaru I., Filimon I., Mirsu O., Tertea I. (1981). Concrete strength and strain. Dev. Civ. Eng..

[4] Muhtar (2019). Experimental data from strengthening bamboo reinforcement using adhesives and hose-clamps. Data in Brief.

[5] Muhtar, Dewi S.M., Munawir A. (2019). The flexural behavior model of bamboo reinforced concrete beams using a hose clamp. Proceedings in Materials Science, Engineering and Chemistry.

[6] Muhtar, Dewi S.M., Wisnumurti, Munawir A. (2018). The stiffness and cracked pattern of bamboo reinforced concrete beams using a hose clamp. Int. J. Civ. Eng. Technol..

